# Optical coherence tomography evaluation of zotarolimus-eluting stents at 9-month follow-up: comparison with sirolimus-eluting stents

**DOI:** 10.1136/hrt.2009.167759

**Published:** 2009-06-16

**Authors:** J-S Kim, I-K Jang, J-S Kim, T H Kim, M Takano, T Kume, N W Hur, Y-G Ko, D Choi, M-K Hong, Y Jang

**Affiliations:** 1 Division of Cardiology, Yonsei Cardiovascular Centre, Yonsei University College of Medicine, Seoul, Korea; 2 Cardiology Division, Massachusetts General Hospital, Harvard Medical School, Boston, Massachusetts, USA; 3 Divison of Cardiology, Nippon Medical School, Tokyo, Japan; 4 Department of Cardiology, Kawasaki Medical School, Kurashiki, Japan; 5 Preventive Medicine, Yonsei University College of Medicine, Seoul, Korea

## Abstract

**Objective:**

To evaluate the vascular response at 9 months after zotarolimus-eluting stent (ZES; Endeavor) implantation using optical coherence tomography (OCT). These findings were compared with those after implantation of a sirolimus-eluting stent (SES; Cypher Select).

**Design:**

Cross-sectional observational study with prospective OCT registry.

**Setting:**

Nine months after ZES or SES implantation.

**Patients and methods:**

A total of 68 patients (32 ZES and 36 SES) underwent OCT at 9 months after stent implantation. The neointima hyperplasia (NIH) thickness inside each strut and percentage of NIH area at every 1 mm cross section were measured.

**Main outcome measurement:**

The degree of neointimal coverage and the prevalence of malapposition at 9 months after ZES and SES implantation using OCT.

**Results:**

The mean (SD) NIH thickness (251.2 (110.0) μm vs 85.5 (53.3) μm, p<0.001) and percentage of NIH area (27.9 (9.1)% vs 11.2 (7.1)%, p<0.001) were significantly greater in ZES than in SES. The prevalence of uncovered strut as well as malapposed strut was significantly lower in ZES than in SES (0.3% vs 12.3%, p<0.001 and 0.08% vs 2.6%, p<0.001). Thrombus was not observed in ZES (0.0% in ZES vs 27.8% in SES, p = 0.001).

**Conclusions:**

Neointimal coverage in ZES was almost complete and malapposition was very rare at 9-months’ follow-up.

Although in-stent restenosis (ISR) was remarkably reduced after introduction of the drug-eluting stent (DES), excessive inhibition of neointima formation caused delayed arterial healing with incomplete endothelialisation over the stent strut.^1^ ^2^ A recent autopsy study showed that the most important histological and morphometric predictors of late stent thrombosis (LST) were endothelial coverage and the ratio of uncovered to total stent struts after DES implantation.^3^ Therefore, identification of neointima over stent struts could provide important information to predict LST. In comparison with the first generation DES, a zotarolimus-eluting stent (ZES) has different inhibition properties for neointimal hyperplasia (NIH).^4^ The ZES has been shown to have a significantly higher rate of neointimal coverage than the sirolimus-eluting stent (SES) on intravascular ultrasound (IVUS) at 8 months after implantation.^5^ However, the resolution of conventional IVUS (100–150 μm) was inadequate to identify neointima over stent struts because a considerable portion of neointima are <100 μm thick.^5^

Intravascular optical coherence tomography (OCT) can identify thin layers of neointima and malapposition with its high resolution (10–20 μm).^6^ ^7^ Several studies have reported that OCT evaluation of stent strut coverage of bare metal stents (BMS) and SES.^6^ ^7^ However, OCT data regarding the degree of neointimal coverage and malapposition after ZES stenting are limited. Therefore, this study was undertaken to evaluate neointimal coverage and malapposition at 9 months after ZES implantation using OCT.

## Material and methods

### Study population

One hundred and seventy-nine patients treated with ZES (Endeavor; Medtronic, Santa Rosa, California, USA) or SES (Cypher Select; Cordis, Miami Lakes, Florida, USA) (90 SES and 89 ZES) who fulfilled inclusion and exclusion criteria were consecutively enrolled in this study between November 2006 and October 2007. The inclusion criteria for this study were: (*a*) de novo lesion with >50% diameter stenosis, which was related to myocardial ischaemia; (*b*) native vessel size of 2.5–3.5 mm in diameter; (*c*) stable angina or acute coronary syndrome (ACS) including ST elevation myocardial infarction; (*d*) non-overlapping stents. The exclusion criteria included: (*a*) significant left main coronary artery disease; (*b*) congestive heart failure or low ejection fraction (⩽35%); (*c*) renal insufficiency with baseline creatinine ⩾2.0 mg/dl; (*d*) lesions unsuitable for OCT with occlusive technique (proximal vessel size >3.5 mm or proximal lesions 15 mm from the ostium of each artery). The choice of DES (ZES or SES) was made according to the operator’s preference. The OCT was performed in 68 patients (32 ZES and 36 SES) during follow-up angiography at 9±2 months. Among 179 patients, we performed a follow-up angiography in 120 patients between 7 and 11 months. Among 120 patients, OCT was performed in only 80 patients because of difficulties and refusal to undergo the OCT procedure. Additionally, 12 stents were not completely evaluated for the whole stent length, and, therefore, we excluded 12 stents after OCT evaluation. The study protocol was approved by the Institutional Review Board of Yonsei University College of Medicine and written consent was obtained from all patients.

### Angiographic analysis

Quantitative coronary angiography (QCA) was performed offline using a quantitative coronary angiography analysis system (CASS; Pie Medical Instruments, Maastricht, The Netherlands) by a single person blinded to patients’ information. Minimal luminal diameter (MLD) of treated coronary segments, reference vessel diameter (RVD), percentage diameter stenosis and lesion length on the baseline angiogram were determined in the view that demonstrated lesions to be most severe and not foreshortened. Post-procedure and follow-up angiograms were evaluated in the same view.

### OCT data collection and analysis

An OCT system (Model M2 Cardiology Imaging System; LightLab Imaging, Westford, Massachusetts, USA) and a 0.014 inch wire-type imaging catheter (ImageWire; LightLab Imaging) were used in this study. A 6 or 7 Fr guiding catheter was introduced into the coronary artery by femoral or radial approach. When the image wire had been positioned through the occlusion balloon distal to the stent, the occlusion balloon (Helios; Avantec Vascular, Sunnyvale, California) was inflated to 0.4–0.6 atm and Ringer’s lactate was infused at 0.5–1.0 ml/s. The image wire was pulled distal to proximal at a speed of 1 mm/s, and continuous images were stored digitally for subsequent analysis. Cross-sectional OCT images were analysed at 1 mm intervals (every 15 frames).

Among 1535 image sections, 1473 image sections (96.0%) were analysable in this study (696/710 (98.0%) in ZES and 777/825 (94.2%) in SES). Parameters were calculated and defined as follows: distances between the endoluminal surface of neointima and stent strut were measured with a measurement line as perpendicular as possible to the neointima and strut.^8^ When there was no definite neointima over the stent strut, we defined it as an uncovered stent strut. Distances between the inner surface of the strut reflection and the vessel wall were measured by extending contours of the walls on the outside of the strut shadow with a measurement line.^8^ The position of the stent strut on the vessel wall was measured by magnifying the individual stent strut to maximise accuracy when the strut was not fully attached to the vessel wall as determined by visual estimation. Stent malapposition was defined as struts with detachment from the vessel wall ⩾160 μm for SES and ⩾110 μm for ZES.^9^ Stent struts at bifurcation with major side branches >2.0 mm in diameter were excluded from this analysis. Thrombus was defined as an irregular mass protruding into the lumen or intraluminal mass unconnected from the surface of vessel wall that had a signal-free shadowing in the OCT image.^10^ Percentage NIH was calculated as percentage NIH area  =  ((stent area − lumen area)/stent area) ×100.

A cross section with uncovered strut was defined if one or more stent struts was uncovered on cross section and a cross section with uncovered strut ratio >0.3 was defined when the ratio of uncovered struts to total stent struts per cross section was more than 0.3.^3^ We identified the variation of NIH thickness and the thickness of NIH at the thinnest and thickest parts on each stent. We also evaluated the pattern for NIH using a heterogeneity score as follows: the status of NIH thickness at each cross section was divided into four grades; grade 0, uncovered strut to total stent struts; grade 1, NIH thickness <100 μm, which was not detected with IVUS; grade 2, NIH thickness between 100 and 200 μm; grade 3, NIH thickness >200 μm.^11^ The grade was determined as minimal and maximal grade including ⩾10% of stent struts at each cross section. The heterogeneity score of NIH thickness in OCT was determined by subtracting the minimal grade from the maximal grade.

### Clinical follow-up

All patients received dual antiplatelet therapy (DAT; aspirin and clopidogrel) for at least 9 months. Death, non-fatal myocardial infarction and LST were evaluated for 9 months.

### Statistical analysis

Inter- and intraobserver variability in measurements of distance and area were assessed by evaluation of 20 random cross-sectional images by two independent readers and by the same reader at two separate time points, respectively. Variations in measurements were calculated using linear mixed models (one- and two-way). Results are expressed as mean (SD) or number (%). Comparisons of categorical variables were made using a χ^2^ test and Fisher exact test while the Student t test was used to compare continuous variables. If the distributions were skewed, a non-parametric test was used. Because of the hierarchical structure of data for all struts across stents and patients, multilevel logistic analysis was applied for uncovered and malapposed struts as the outcome variable to control for the random and fixed effects of the strut, lesion and patient characteristics. At the lesion level, lesion type (A, B1 or B2, C), stent type (ZES vs SES), stent length, stent diameter, RVD, MLD and maximum inflation pressure were considered. At the patient level, age, sex, ACS and diabetes mellitus were considered.

All analyses were performed using Statistical Analysis Software (SAS) 9.1.3. (SAS Institute, Cary, North Carolina, USA). A p value <0.05 was considered statistically significant.

## Results

### Baseline characteristics

Table 1 presents the baseline characteristics of patients. Although DES were not randomly selected, baseline characteristics were similar between the two stents groups. The mean (SD) interval between stent implantation and OCT was also similar between the two groups (289 (59) days in ZES vs 288 (69) days in SES, p = 0.95). The prevalence of ACS was 55.9% in all patients and the left anterior descending artery was the most commonly treated lesion in both groups (15 (46.9%) in ZES vs 18 (50.0%) in SES, p = 0.41).

**Table 1:** Baseline characteristics and quantitative coronary angiography findings

Baseline characteristics	ZES (n = 32)	SES (n = 36)	p Value
Age (years)	59.7 (8.4)	59.6 (8.4)	0.95
Male, n (%)	25 (78.1)	25 (69.4)	0.42
Acute coronary syndrome, n (%)	17 (53.1)	21 (58.3)	0.67
Hypertension, n (%)	17 (53.1)	15 (41.7)	0.35
Diabetes mellitus, n (%)	12 (37.5)	13 (36.1)	0.91
Hyperlipidaemia, n (%)	18 (56.3)	17 (47.2)	0.46
Smoking, n (%)	8 (25.0)	10 (27.8)	0.50
Chronic total occlusion, n (%)	0 (0.0)	1 (2.8)	1.00
Target vessel			0.33
LAD, n (%)	15 (46.9)	17 (47.2)	
LCX, n (%)	4 (12.5)	8 (25.0)	
RCA, n (%)	13 (40.6)	10 (27.8)	
Lesion type B2 or C, n (%)	24 (75.0)	28 (77.8)	0.79
Stent diameter (mm)	3.1 (0.3)	2.9 (0.3)	0.004
Stent length (mm)	23.7 (5.9)	24.1 (6.4)	0.78
Maximal pressure (atm)	17.1 (2.4)	17.2 (2.0)	0.17
			
*Postintervention QCA data*			
Mean RVD (mm)	2.9 (0.3)	2.8 (0.4)	0.32
MLD (mm)	2.8 (0.3)	2.7 (0.4)	0.04
DS (%)	2.0 (5.9)	5.9 (7.0)	0.02
Acute gain (mm)	2.1 (0.5)	2.0 (0.5)	0.33
			
*9 Months’ follow-up QCA data*			
Mean RVD (mm)	2.7 (0.3)	2.7 (0.4)	0.79
MLD (mm)	2.3 (0.4)	2.5 (0.4)	0.06
DS (%)	15.1 (8.2)	8.6 (13.4)	0.02
Late loss (mm)	0.5 (0.3)	0.2 (0.5)	<0.001

Values are presented as mean (SD) unless stated otherwise.

DS, diameter stenosis; LAD, left anterior descending artery; LCX, left circumflex artery; MLD, minimal lesion diameter; QCA, quantitative coronary angiography; RCA, right coronary artery; RVD, reference vessel diameter; SES, sirolimus-eluting stent; ZES, zotarolimus-eluting stent.

The mean (SD) stent diameter was larger in ZES than SES (3.1 (0.3) mm vs 2.9 (0.3) mm, p = 0.004), but stent length and lesion type were similar in both groups (table 1). Maximal balloon inflation pressure was 17.1 atm in ZES and 17.2 atm in SES without significant difference in both groups (table 1).

### Angiographic data

Preintervention QCA data were similar between the two groups (mean (SD) RVD: 2.9 (0.3) mm in ZES vs 2.8 (0.4) mm in SES, p = 0.25 and MLD: 0.8 (0.5) mm in ZES vs 0.7 (0.4) mm in SES, p = 0.50). Although acute gain was similar between the groups, both diameter stenosis and late loss at 9 months after implantation were significantly greater in ZES (table 1).

### OCT data

**Figure 1:**
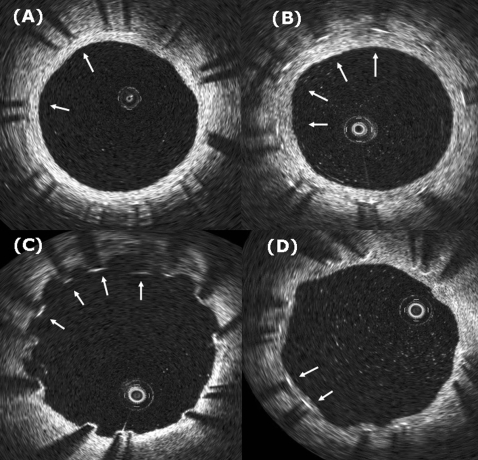
Representative optical coherence tomography images of zotarolimus-eluting stents (ZES) and sirolimus-eluting stents (SES). Completely covered struts (A) and thick neointima (B) were seen in ZES but malapposed and uncovered struts (C) and thin neointima (D) were seen in SES.

Table 2 shows the OCT data. Total measured stent length was 1535 mm including 16 563 struts (8497 in ZES and 8066 in SES). Figure 1 shows representative OCT cases in ZES and SES. Analysable stent struts for each cross section was greater in ZES than SES (12.2 struts (8497/696) in ZES and 10.4 struts (8066/777) in SES). The mean (SD) stent area was significantly larger in ZES than in SES (7.8 (1.7) mm^2^ vs 6.6 (1.6) mm^2^, respectively, p<0.001), which was consistent with QCA. Additionally, NIH area (2.2 (0.8) mm^2^ vs 0.7 (0.4) mm^2^, p<0.001) and percentage of NIH area (27.9 (9.1)% vs 11.2 (7.1)%, p<0.001) were greater in ZES than in SES. The intraobserver correlation coefficient of neointimal thickness or distance and area between variability of a single observer was 0.981 (95% CI 0.975 to 0.985) and 0.997 (95% CI 0.993 to 0.999) using a one-way mixed model, in which patient effects are random. The interobserver correlation coefficient of neointimal thickness or distance and area between the variability of two observers using a two-way mixed model, in which patient effects were random and observers’ effects were fixed, was 0.982 (95% CI 0.977 to 0.986) and 0.993 (95% CI 0.982 to 0.997).

**Table 2:** Optical coherence tomography (OCT) measurements at 9-months’ follow-up

Tomography measurements	ZES (n = 32)	SES (n = 36)	p Value
Cross sections (n)	696	777	–
Mean stent area (mm^2^)	7.8 (1.7)	6.6 (1.6)	0.006
Mean lumen area (mm^2^)	5.6 (1.5)	6.1 (1.7)	0.35
Mean NIH thickness (μm)	251.2 (110.0)	85.5 (53.4)	<0.001
Mean NIH area (mm^2^)	2.2 (0.8)	0.7 (0.4)	<0.001
Percentage of NIH	27.9 (9.1)	11.2 (7.1)	<0.001
Heterogeneity score	0.9 (0.8)	1.1 (0.7)	<0.001
Cross section with uncovered strut(s), n (%)	18 (2.6)	308 (39.6)	<0.001
Cross section with uncovered strut ratio >0.3, n (%)	0 (0.0)	140 (18.0)	<0.001
Presence of thrombi, n (%)	0 (0.0)	10 (27.8)	0.001

Values are presented as mean (SD), unless stated otherwise.

Heterogeneity score was defined as the difference between maximal and minimal neointimal grade. (The status of NIH thickness at each cross section was divided into four grades; grade 0, uncovered strut to total stent struts; grade 1, NIH thickness <100 μm, which was not detected with intravascular ultrasound; grade 2, NIH thickness between 100 and 200 μm, which was between minimal thickness detectable using OCT and minimal mean thickness in bare metal stents; grade 3, NIH thickness over 200 μm.^11^ The grade was determined as minimal grade including ⩾10% of stent struts at each cross section.)

NIH, neointima hyperplasia; SES, sirolimus-eluting stent; ZES, zotarolimus-eluting stent.

**Figure 2:**
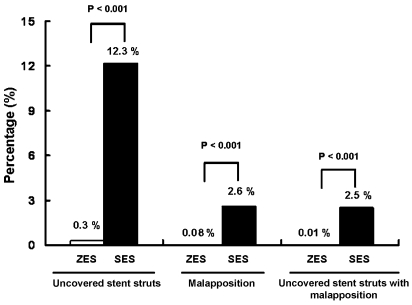
Difference in the rate of uncovered stent strut, malapposition and uncovered stent strut with malapposition on optical coherence tomography between zotarolimus-eluting stents (ZES) and sirolimus-eluting stents (SES).

In a comparison of neointima over the stent struts of ZES and SES, it was found that most stent struts of ZES were covered with neointima. However, >10% of stent struts in SES were not covered with neointima (uncovered stent struts of ZES vs SES: 28/8497 (0.3%) vs 991/8066 (12.3%), p<0.001, fig 2). The rate of malapposition (7/8497 (0.08%) vs 208/8066 (2.6%), p<0.01) and uncovered stent strut with malapposition (1/8497 (0.001%) vs 199/8066 (2.5%), p<0.001) were significantly higher in SES (fig 2). Cross sections with uncovered struts were detected more frequently in SES than ZES. In particular, there were no cross sections with an uncovered strut ratio >0.3 in ZES compared with 140 (18.0%) cross sections in SES. Intracoronary thrombus within stents was also not detected in ZES (10 (27.8%) in SES vs 0 (0.0%) in ZES, p = 0.001) (table 2). The uncovered (281/2524 (11.1%) vs 738/14 039 (5.3%), p = 0.010) and malapposed struts (96/2524 (3.8%) vs 119/14 039 (0.8%), p<0.001) were frequently found in the stents with intracoronary thrombus

**Figure 3:**
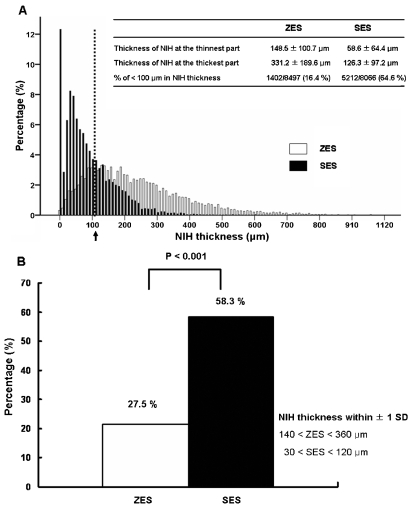
Statistical distribution of neointimal hyperplasia (NIH) thickness of zotarolimus-eluting stents (ZES) and sirolimus-eluting stents (SES) (A). The proportions of NIH thickness beyond 1 SD between ZES and SES. The percentage of NIH thickness outside 1 SD was significantly lower in ZES than SES (B). The thickness of NIH at the thickest part or thinnest part was the mean NIH thickness at three consecutive cross sections at the corresponding part.

NIH pattern and the mean neointima thickness were quite different between the two stent groups (fig 3A). The thickness of NIH at the thinnest part in ZES was similar to that at the thickest part in SES. The proportion of NIH thickness beyond 1 SD was significantly lower in ZES (27.5% vs 58.3%) (fig 3B). The heterogeneity score was also significantly lower in ZES (table 2).

### Predictors of uncovered and malapposed stent struts

Table 3 shows predictors of uncovered and malapposed struts. The stent type, presence of a lesion typeB2 or C and diabetes mellitus were independent predictors of uncovered struts, while stent type and diabetes mellitus were independent predictors of malapposed struts, respectively.

**Table 3:** Univariate and multivariate predictors of uncovered and malapposed struts

Predictors	Odds ratio (95% CI)	p Value
Univariate predictors of uncovered struts		
Stent type: ZES	0.89 (0.84 to 0.93)	<0.001
Lesion type B2 or C	1.07 (1.01 to 1.13)	0.03
Acute coronary syndrome	1.06 (1.01 to 0.13)	0.03
Diabetes mellitus	1.05 (0.99 to 0.12)	0.07
		
Multivariate predictors of uncovered struts		
Stent type: ZES	0.87 (0.82 to 0.91)	<0.001
Lesion type B2 or C	1.10 (1.02 to 1.20)	0.02
Diabetes mellitus	1.06 (1.00 to 1.12)	0.04
		
Univariate predictors of malapposed struts		
Stent type: ZES	0.98 (0.97 to 0.99)	0.01
Minimal luminal diameter before PCI	0.99 (0.96 to 1.00)	0.06
Acute coronary syndrome	1.02 (1.00 to 1.04)	0.09
Diabetes mellitus	1.03 (1.00 to 1.05)	0.09
		
Multivariate predictors of malapposed struts		
Stent type: ZES	0.97 (0.95 to 0.99)	0.005
Diabetes mellitus	1.03 (1.01 to 1.05)	0.004

PCI, percutaneous coronary intervention; ZES, zotarolimus-eluting stent.

### Clinical outcome

Follow-up angiography at 9 months was available in 120 patients (62 ZES and 58 SES). ISR occurred in eight and five patients in the ZES and SES group, respectively. But, OCT examination was possible in only four patients treated with SES among 13 patients with ISR. In both groups there were no deaths, non-fatal myocardial infarction and LST during the 9 months.

## Discussion

Our study showed that neointimal coverage of stent struts was almost complete with a more homogeneous pattern in ZES at 9 months. Uncovered and malapposed struts were frequently observed and the NIH pattern was more heterogeneous in SES.

A previous pathological report demonstrated an association between the lack of neointimal coverage in stent struts and thrombus formation.^12^ Additionally, several angioscopy studies demonstrated that thrombi were more prevalent in SES without complete neointimal coverage than in BMS.^13^ ^14^ Therefore, detection of neointima of stents might have an important clinical implication for predicting LST and deciding the optimal duration of DAT.

Several imaging modalities can be used to study neointimal coverage of stents. IVUS has been widely used to detect NIH after stent implantation, but it has a critical limitation in resolution that can only differentiate neointima thicker than 100 μm. Therefore, it is impossible to evaluate precisely neointimal coverage in DES because previous studies, including this study, have shown that a considerable portion (40%) of neointima is <100 μm in thickness.^6^ ^8^ Angioscopy might be a useful tool to evaluate neointima by providing direct visualisation, but it is limited by the unobtainability of quantitative information.^15^

Recently, OCT has been introduced as a high-resolution imaging modality with 10–20 μm of axial resolution.^16^ ^17^ The new intravascular imaging tool could clearly detect thin neointima and provide quantitative information. Miyazawa *et al* reported the rates of neointimal coverage were 50.2% in ZES and 10.5% in SES at 8 months but other OCT data for SES showed 85–91% of neointimal coverage between 3 and 6 months.^6^ ^8^ ^15^ Recently, Guagliumi, *et al* also demonstrated that both ZES and SES had over 90% of neointimal coverage at 6 months.^18^ Our study produced a similar result showing that 88% of struts were covered by neointima. In addition, our study showed that two-thirds of stent struts in SES and about 40% in ZES were <100 μm in neointimal thickness (fig 2A). These findings imply that IVUS analysis for neointima might be inaccurate owing to the relatively poor resolution. A large portion of neointima thickness was <100 μm on ZES as well as SES. However, OCT can measure precisely the NIH thickness compared with histology, which was demonstrated on a rabbit carotid stent model (r = 0.85, p<0.001).^19^

This study showed that neointima covered almost all of the stent struts and was evenly distributed in ZES compared with that in SES (99.7% in ZES vs 87.7% in SES). This finding was similar to previous angioscopy findings between SES and ZES and compatible with those of BMS.^5^ ^8^ ^15^ ^20^ The ODESSA study showed that ZES had a significantly lower incidence of uncovered stent struts than SES in both overlapping and non-overlapping sites (0.02 vs 5.8% in an overlapping site and 0.01% vs 6.0% in a non-overlapping site).^18^ Although these OCT and angioscopy findings suggested that DAT for 9 months might be acceptable in ZES, healthy neointima with intact functional endothelium could be crucial to predict LST in the future and the optimal duration of DAT. In addition, there were no data suggesting that neointimal coverage detected by OCT is related to clinical events or guidance for duration of DAT. Thus, more data and longer-term follow-up are needed to investigate the clinical implications.

A previous pathology study showed that non-uniform neointimal coverage with DES, which was indicated by the number of uncovered struts for each cross section, markedly increased stent thrombosis risk, and lack of neointimal coverage was associated with thrombus formation.^3^ Therefore, heterogeneous coverage of neointima over stent struts might also be related to future thrombotic events. Our data demonstrated that the absolute percentage of neointimal coverage was higher in ZES than in SES, and ZES also has a homogeneous pattern of neointimal coverage over stent struts. Analysable stent struts were greater in ZES than SES, which might be related to fewer artefacts around struts because of evenly covered neointima in ZES. In addition, cross sections with uncovered struts were significantly less frequently detected in ZES, and, significantly, the ratio of cross sections with uncovered to total stent struts >0.3 was not found in ZES, which was the morphometric predictor of LST in the autopsy study.^3^

Malapposition is another possible predictor of stent thrombosis, though there has been some disagreement in the literature.^21^ ^22^ High-resolution OCT could be used to determine the presence of malapposition. In this study, SES showed a significantly higher rate of malapposition than ZES. Malapposition in SES might be related to neointima growth over stent struts by some magnitude but the clinical relevance was not clear and more investigation is needed.

### Study limitations

This study had several limitations. First, it was a non-randomised study and the initial OCT images immediately after implantation were not available. Second, this study had a relatively small population who had a follow-up angiography and OCT, which might have resulted in selection bias. However, the number of stent struts (more than 15 000 struts) was enough to evaluate neointima or malapposition. Third, the quality as well as quantity of NIH might be important to prevent thrombus formation. We could not evaluate the quality of neointima in this study.

## Conclusions

ZES, compared with SES, showed an almost complete homogeneous neointimal coverage of stent struts and low rate of malapposition at 9 months after implantation.
